# Using intersectionality to inform fit-for-purpose patient-reported outcome measure development: a qualitative interview study

**DOI:** 10.1186/s41687-026-01132-y

**Published:** 2026-07-24

**Authors:** Tilly Stott, Debbie Cavers, Kayla Ostrishko, Julia Lawton

**Affiliations:** 1 Mapi Research Trust, Lyon, France; 2 Usher Institute and Edinburgh Medical School, University of Edinburgh, Edinburgh, UK

**Keywords:** Intersectionality, Patient-reported outcome measure (PROM), Patient-reported outcome measure (PROM) development, Fit-for-purpose measurement, Qualitative research

## Abstract

**Background:**

Patient-reported outcome measures (PROMs), often self-report questionnaires, capture a patient’s health status as reported directly by the patient. PROMs are increasingly being used to collect patients’ perspectives regarding health outcomes influencing their treatment and broader decisions about intervention efficacy, reimbursement, and service provision. Concerns are growing about how fit-for-purpose many PROMs are, with implications for health inequity. Health researchers are starting to explore how intersectionality can be used to understand health inequity, but intersectionality is rarely discussed in PROM development literature. This study sought to understand and explore PROM researchers’ views about using intersectional approaches to develop fit-for-purpose PROMs.

**Methodology:**

Semi-structured interviews were conducted online with PROM researchers (*n* = 8) who both had and had not previously engaged with intersectionality. Interviews focused on perceived barriers to using intersectionality to inform PROM development and how such barriers could be overcome. With consent, interviews were recorded, transcribed and analysed using a reflexive thematic approach.

**Results:**

Participants reported limited understanding about intersectionality in the health outcomes field and how it can inform more fit-for-purpose PROM development. Concerns about methodological rigour in PROM development were also cited with participants noting that if existing shortcomings in how PROMs are developed are not recognised, it would be challenging to persuade researchers to change practices. Another challenge raised was reaching certain groups to participate in research, partly due to mistrust of medical research and healthcare professionals. Participants also expressed concerns about financial costs, noting that intersectional approaches may entail bigger budgets and longer study timelines. Participants likewise highlighted the role of the socio-political context in influencing what researchers could feasibly achieve. Interviewees from both groups proposed approaches to address barriers. These included garnering more diverse perspectives from research participants through community-based participatory research and establishing more diverse PROM development teams. They emphasised the value of clearly defining responsibility for instigating change. Participants finally outlined key arguments to help encourage stakeholders to support more intersectional approaches.

**Conclusions:**

Findings from this exploratory study suggest that despite considerable barriers, some PROM developers view intersectionality as a valuable framework for addressing existing PROM shortcomings. We provide tentative recommendations for encouraging PROM developers to use intersectionality to inform PROM development and measure health outcomes that are more meaningful to patients.

**Supplementary Information:**

The online version contains supplementary material available at https://doi.org/10.1186/s41687-026-01132-y.

## Abstract (plain language summary)

When health professionals and clinical researchers make decisions, it is important to take into account what matters most to patients. One way to do this is by using questionnaires called patient-reported outcome measures (PROMs), which ask patients about their health and experiences. However, if these questionnaires aren’t designed with input from a wide range of patients, they may miss important perspectives. This means some patient voices will not be heard when decisions are made. The idea of intersectionality looks at how different aspects of identity like race, gender, and social background interact to shape experiences. This approach is often used in the social sciences but rarely in health research when creating patient questionnaires. Our study explored why intersectionality is not widely used in this area, what challenges exist in doing so, and how to overcome them. We interviewed researchers who design these questionnaires and found several barriers. These barriers included PROM researchers not understanding what intersectionality means or how it can help create better questionnaires, difficulties reaching diverse patients to help design the questionnaires, and pushback because of budgetary or socio-political concerns. Our participants also suggested practical ways to help overcome these challenges. These included bringing in more diverse viewpoints (from patients and researchers), deciding who should lead the changes, and presenting arguments to convince researchers to change. Based on our findings, we present suggestions to help researchers include intersectional thinking when developing PROMs.

**Supplementary Information:**

The online version contains supplementary material available at https://doi.org/10.1186/s41687-026-01132-y.

## Background

Patient-reported outcome measures (PROMs) are a type of Clinical Outcome Assessment (COA) defined as a “measure that describes or reflects how a patient feels, functions, or survives” [1]. PROMs are typically self-report questionnaires that capture the “status of a patient’s health condition that comes directly from the patient” without external interpretation [2]. They commonly assess symptoms, physical or psychological functioning, or health-related quality of life [3]. PROMs provide a standardised means of measuring patient perspectives and are widely used to inform regulatory decisions on treatment approval [4], clinical decision-making [5–6], service-provider performance evaluation [7], and cost-effectiveness analyses [8].

The growing use of PROMs has driven the development of thousands of instruments, making PROM selection challenging for researchers [9]. In response, over 40 guidelines [10] have been published outlining key characteristics to consider when developing or selecting PROMs. More recently, the US Food and Drug Administration’s (FDA) Patient-Focused Drug Development (PFDD) guidelines [11] have shifted attention towards assessing how ‘fit-for-purpose’ a PROM is. For the FDA, ‘fit-for-purpose’ refers to “the strength of the evidence in support of interpreting the COA scores as reflecting the concept of interest within the context of use” [11].

While efforts such as the FDA’s PFFD guidelines have sought to improve PROM quality, concerns persist regarding how fit-for purpose many PROMs are for truly capturing and representing the diversity of patient experiences. Oluloro reported that numerous PROM items used in gynaecologic cancer trials lacked relevance for Black women [12]. Similarly, Nikolovski highlighted that many PROMs fail to “reflect the priorities and lived experiences of people who are CALD [people from culturally and linguistically diverse backgrounds] or Indigenous Peoples”, thereby contributing to poor PROM uptake [13]. Ramirez further warned that PROMs may not adequately capture heterogeneous patient experience across populations if they are not designed to “address the racial, ethnic, gender, cultural, and inclusivity needs of the patient populations that they aim to serve” [14]. Calvert likewise noted limitations to PROMs’ ability to measure disease impact, symptoms, or QoL [quality of life] when data are unrepresentative [15]. These concerns all relate to how-fit-for-purpose a PROM can be for capturing outcomes in diverse populations, as individuals “may not interpret and respond to questions about their health and their QoL in the same way” [16].

It has been suggested that adopting an intersectional lens may help to better understand the inadequacies of PROMs [16], which in turn could facilitate the integration of a broader spectrum of perspectives within rigorous PROM development. Intersectionality is a term with a long and complex history [17–21]. According to Collins, it can be thought as offering the:

*Critical insight that race*,* class*,* gender*,* sexuality*,* ethnicity*,* nation*,* ability*,* and age operate not as unitary*,* mutually exclusive entities*,* but as reciprocally constructing phenomena that in turn shape complex social inequalities.* [22].

Collins’ definition highlights how intersectionality considers the coalescence of identities that inform experience and how this intersection can subsequently result in social inequalities. Social inequalities in health can be understood as the differences in health at individual and population levels [23]. When these inequalities are unfair, avoidable, and systematic, such disparities are termed health inequities [23–24]. Intersectionality can serve as a framework for understanding how individuals experience inequity multidimensionally [25].

To date, few PROM developers have published studies explicitly discussing intersectionality in relation to PROM development (see additional materials). Furthermore, whilst there are numerous papers on best practices for developing PROMs [10], there is no clear guidance on how intersectionality can be used to inform PROM development. To begin to address this unmet need, we conducted an exploratory study to understand researchers’ views about using intersectional approaches to develop fit-for-purpose PROMs. Our central research question was: what do researchers think the barriers and facilitators are to using intersectionality to inform PROM development? Our objective was to provide recommendations to support the use of intersectional approaches to inform PROM development.

## Methods

### Study design

Given the study’s exploratory nature, we used a qualitative design, aligned with an interpretivist approach, comprising semi-structured interviews with a group of PROM experts. This design allowed us to address our study aim while affording participants the flexibility to raise unforeseen issues they considered salient. We used an iterative approach to data collection and analysis, wherein topic guides were revised throughout data collection to allow initial findings to inform the areas explored in later interviews.

### Positionality statement

The study was initiated by TS (she/her), a white woman trained in public health and the humanities. Her interest in the limited uptake of intersectionality in PROM development and health outcomes research was informed by her use of intersectionality as a framework for understanding how historical and structural processes shape contemporary inequities. This interest was complemented by JL’s (she/her) anthropological training, DC’s (she/her) health psychology training, and KO’s (she/they) training in psychology and population health.

As four white researchers, we acknowledge that race influenced how we engaged with the topic of intersectionality and constructed meaning from participants’ accounts. Accordingly, we engaged in reflexive discussion about how our social, racial, and disciplinary positions informed the research process, including the framing of questions, interpretation of participants’ narratives, and presentation of findings. Together, this shaped our approach to health outcomes research which was attentive to the broader social and historical contexts.

### Recruitment and sampling

Our sampling decisions were informed by an initial literature review and TS’s professional experience as a COA specialist.

We recruited PROM researchers who had and had not engaged with intersectionality, designated as Groups A and B respectively. We defined PROM researchers as individuals with experience developing a PROM, and engagement with intersectionality as having published work in which the authors refer to the term “intersectionality”. This broad definition was chosen in recognition of the small number of PROM researchers who have published work referring to intersectionality. In making this decision, we recognised that some PROM researchers might have been familiar with intersectionality or had adopted an intersectional approach in their research without having explicitly published work that referenced this concept. However, we considered our classification the most effective way of identifying PROM researchers actively engaged in intersectionality-informed PROM research.

We included researchers who had not engaged with intersectionality (Group B) to allow us to explore potential differences in the perceived barriers and facilitators to engagement (as compared to Group A participants). Group B’s primary inclusion criteria presented a larger pool of potential participants since most PROM researchers have not explicitly engaged with intersectionality in the published literature. To encourage a range of perspectives within Group B, we developed a set of ideal attributes with participants needing to meet at least one. A summary of the inclusion and exclusion criteria is presented in Table 1.

**Table 1:** Inclusion and exclusion criteria

	Group A: PROM researchers who have engaged with intersectionality	Group B: PROM researchers who have not engaged with intersectionality
Inclusion criteria	Has explicitly engaged with intersectionality in the published literature and developed a PROM.	Has experience with PROM development.
		*Additional ideal attributes*: • Extensive experience developing PROMs. *Rationale: To understand what the reasons*,* and potential barriers*,* might have been to not explicitly engaging with intersectionality in the published literature despite having developed many PROMs.* • Published work on diversity, equity, and inclusion (DEI). *Rationale: To compare the differences in responses between researchers interested in DEI and researchers explicitly publishing on intersectionality.* • Developed a PROM qualified by the FDA COA Qualification Program. *Rationale: This program is focused on COAs “intended to address unmet public health needs” and a qualified COA is considered to be a “well-defined and reliable assessment” *[26].* We thought it would be insightful to explore the perspective of PROM developers who had participated in this rigorous process.*
Exclusion criteria	Does not speak English.	Does not speak English.
	Has no experience with PROM development.	Has no experience with PROM development.

To identify potential participants, we reviewed the intersectionality and PROM development literature. We then drew up a preliminary list of potential participants whose names were in the public domain and we matched them against the inclusion criteria. TS shared this list with PROM experts in her network and, based on their feedback, one more potential participant was added. We also used snowballing, wherein participants were asked to suggest other researchers who met our inclusion criteria. Thirty-two researchers were informed about the study by email and invited to opt-in by emailing TS.

### Data collection

The interview topic guide was informed by PROM development and intersectionality literature, and discussions with two PROM experts and an intersectionality academic. Pilot interviews were conducted by TS with two further PROM experts, known to her through her personal networks, to test out and refine the contents.

Interviews (53–69 min) were conducted by TS between January and March 2025 using Microsoft Teams with audio recordings and transcripts generated. Using the topic guide as a foundation, TS asked open-ended questions to elicit a broad range of responses. For further information about the main areas explored, see additional materials.

### Analysis

Data were analysed using a reflexive thematic approach [27–31]. The analysis was led by TS in close collaboration with JL and with input from DC (JL and DC are female researchers with over 50 years of collective experience of conducting and analysing in-depth interviews). The analysis commenced through data familiarisation, which entailed listening to interview recordings as well as repeated read throughs of all transcripts. This enabled the identification of preliminary themes which were refined through conversations with JL, although umbrella themes (facilitators and barriers) were pre-defined based on our research question.

Next, the transcripts were imported into NVivo14 [32]. We generated codes using participants’ own words and gerunds to reflect their agency in the narrative [33]. Cross-cutting themes were generated by comparing interviews and through discussion between TS and JL, which resulted in a framework for data coding. We then established patterns connecting the data to create code clusters (sub-themes) where appropriate, which were collated under themes. Themes and sub-themes were reviewed for coherence and consistency across the whole dataset to establish our final thematic model. We employed a reflexive approach throughout using journalling and regular discussion to encourage active awareness of how our positionality as researchers influenced our study design, data collection, analysis, and interpretation of the findings.

### Ethics

This study was approved by the University of Edinburgh’s Usher Masters Research Ethics Group (UM241109/08-Jan-2025). This publication follows the consolidated criteria for reporting qualitative research (COREQ) checklist (see additional materials) [34].

### Findings

#### Participant demographics

Eight PROM researchers who worked in a variety of contexts, including clinical trials, hospitals, government programs, and clinical practice were interviewed. They were based in the United States of America (USA), United Kingdom (UK), Canada, Brazil, and Australia/France. An overview of interviewees’ groups and background is presented in Table 2; however, demographic information has been kept to a minimum to protect participants’ identities.

**Table 2:** Participant Demographics

	Group*	Sector Type
Participant one	B	Academic/Public sector
Participant two	A	Public sector
Participant three	A	Academic/Public sector
Participant four	B	Academic
Participant five	B	Private sector
Participant six	A	Private sector/Public sector
Participant seven	B	Private sector
Participant eight	B	Private sector

*Group A: PROM researcher who has explicitly engaged with intersectionality in published literature

*Group B: PROM researcher who has not explicitly engaged with intersectionality in published literature

**Fig. 1:**
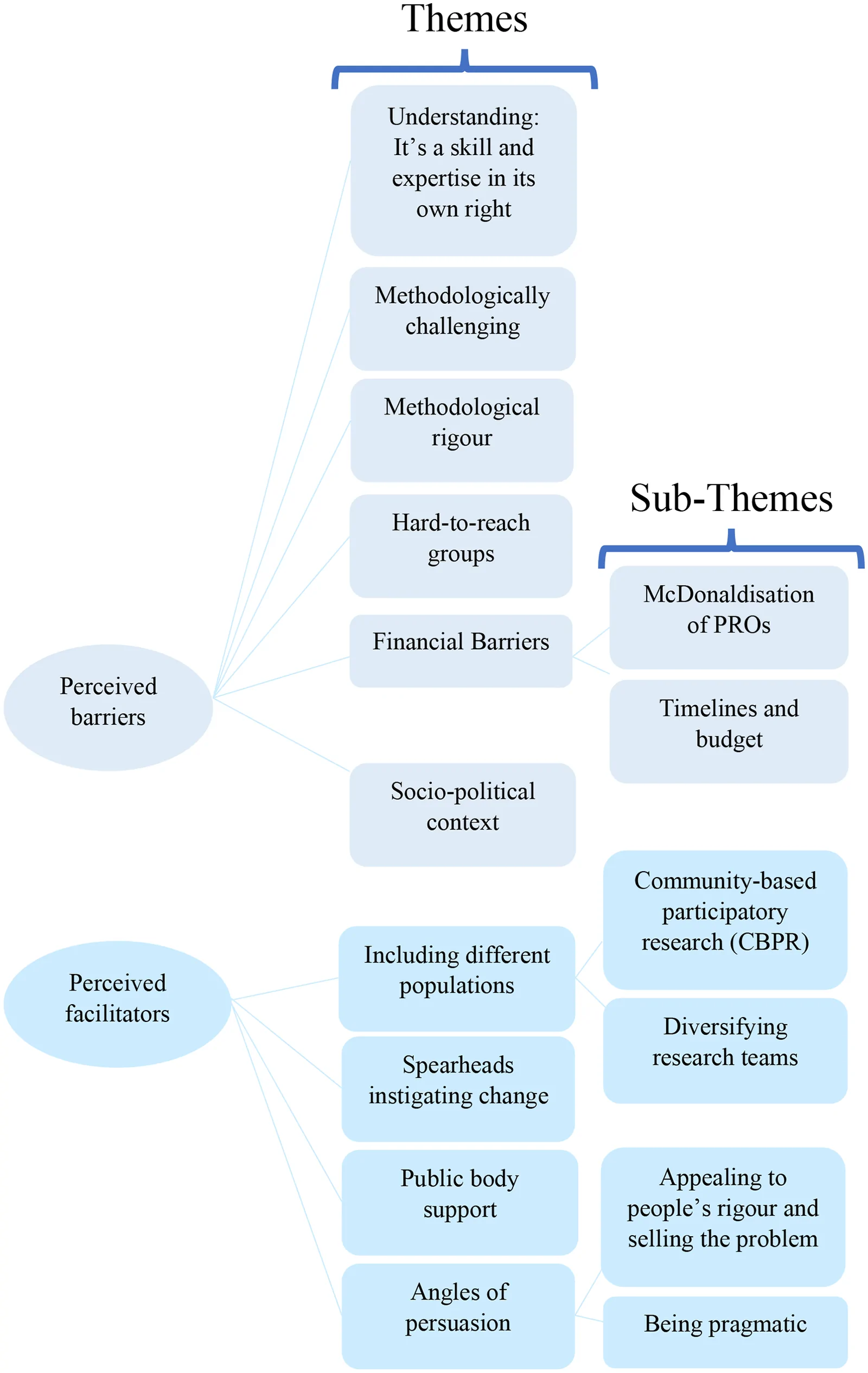
Summary of themes and sub-themes of what researchers perceive the barriers and facilitators to be to using intersectionality to inform PROM development

A summary of themes and sub-themes, classified under perceived barriers and facilitators, is presented in Fig. 1. These are reported in more detail below. Supporting illustrative quotes are presented in Tables 3 and 4.

**Table 3:** Perceived barriers to adopting intersectional approaches to inform fit-for-purpose PROM development – Supporting Illustrative Quotes

Descriptions	Supporting Illustrative Quotes
**Theme: Understanding: It’s a skill and expertise in its own right**	
Understanding of intersectionality	Quote 1: *“I always view it like Venn diagrams*,* intersectionality… these identities [are] like overlapping concentric circles.”* (Participant Six - Group A – Private sector/Public sector)
	Quote 2: *“Understand*,* you know*,* general principles.”* (Participant Seven - Group B - Private sector)
	Quote 3: *“So has it been thought about? Yes. Has it been talked about? To a degree.”* (Participant Eight - Group B - Private sector)
Lack of understanding about what intersectionality is amongst colleagues and the wider field	Quote 4: “*…they [the research team] went with the idea but they didn’t understand like the kind of nuts and bolts of bolts of it [intersectionality].*” (Participant Four - Group B - Academic/Public sector)
Impact of academic background on how well intersectionality is understood	Quote 5: “*I feel that they [with the social sciences background] were the ones that were like more leading this process*,* knowing how we could do that… [the]team with more experience on quantitative research… [were] less familiar with [intersectionality].”* (Participant Two - Group A - Academic/Public sector)
	Quote 6: “*But it wasn’t until I got to university that*,* that [intersectionality] was something that I was taught*,* like how to think about it*,* or how to even talk about it*.” (Participant Six - Group A – Private sector/Public sector)
**Theme: Methodologically challenging**	
	Quote 7: *“As with a lot of researchers*,* still struggling a little bit to make sure we can incorporate some of that [intersectionality] in the way that we approach small sample qualitative research.”* (Participant Seven - Group B - Private sector)
	Quote 8: *“There’s a lot to be said about marrying the principles of including diverse viewpoints and methodologically fitting all of those concepts within one measure… because by definition*,* you’re going to get divergence. And how do you manage that within a single tool? I think that’s very*,* very difficult.”* (Participant Four - Group B - Academic/Public sector)
**Theme: Methodological rigour**	
	Quote 9: “*While the FDA sets out all of these criteria*,* not everyone is adhered to FDA and like the use of PROs are far broader than in micro-regulatory and drug development space. I mean*,* you have people just using the… I’m pointing over there because there’s our local hospital there… like there are clinicians who develop their own PROs like all the time*.” (Participant Four - Group B - Academic/Public sector)
	Quote 10: *“[There are] cottage industries of academics who don’t know how to make a questionnaire putting out a new questionnaire and saying it’s… ‘valid’… it’s just so wrong*,* they so don’t understand what’s going on… a questionnaire is never validated… it’s just profound lack of understanding and methodological training.”* (Participant One - Group B - Academic/Public sector)
	Quote 11: *“We don’t view a tool as valid or not. Validity is no longer a property of the tool… Validity is an expression of whether our inferences arising from the use of that tool are warranted*,* and that shift is taking hold…. [but] there’s still a lot of*,* you know*,* writing about “this tool is valid or not valid”*,* so it’s*,* it’s not a complete shift.”* (Participant Three - Group A - Academic/Public sector)
	Quote 12: *“It’s not being made explicit when you’re writing proposals*,* writing your findings*,* anything like that. But this is something that for like would say other paradigms and within which most qualitative studies are aligned with. This is something that is part of doing the research the study. So*,* you’re*,* you’re stating your epistemological stance*,* stand*,* and you’re analysing implications of that and your values and things…”* (Participant Two - Group A - Academic/Public sector)
**Theme: Hard-to-reach groups**	
Difficulties finding certain groups	Quote 13: *“I guess the barrier is acknowledging those people might be harder to find*,* especially if it is*,* you know*,* an intersection between they’re less common to have that condition*,* but then they’re also not as represented in that country of interest.”* (Participant Five - Group B - Private sector)
	Quote 14: *“There are certain disease areas that are just very difficult to recruit any patients… we’re lucky enough to be able to cut our groups by*,* you know*,* one factor*,* let alone understand all of the different ways in which you know*,* race and education and comorbidities and all of these other things kind of play together.”* (Participant Seven - Group B - Private sector)
Mistrust of the medical field	Quote 15: *“I don’t feel confident that the black woman I’m talking to in a research study is sharing with me the same experience of her disease and her journey as the black woman who is afraid*,* or has barriers to participating in a research study because of*,* you know*,* Tuskegee in the history*,* right? So*,* there’s a huge amount of discomfort with the establishment and research in general.”* (Participant Eight - Group B - Private sector)
**Theme: Financial Barriers (Sub-theme McDonaldisation of PROs)**	
	Quote 16: *“There’s a lot of ‘McDonaldisation’ of PROs. It’s an entire industry which is tremendously profit making… I don’t see anything that’s going to stop the industry… I don’t see anything can stop and improve it… there’s a very big industry to keep the status quo.”* (Participant One - Group B - Academic/Public sector)
**Theme: Financial Barriers (Sub-theme Timelines and Budgets)**	
	Quote 17: *“Within pharma*,* within industry*,* it’s driven by value and profit*,* which is sometimes how much money you’re putting in*,* is often how much time something takes.”* Quote 18: *“It’s already really difficult to fight for patient-centred research in a pharmaceutical company. If I were to add onto that*,* I need more time*,* more money to be more inclusive and not inclusive for inclusivity’s sake but inclusive to be more scientifically appropriate and accurate to have a more scientific approach. The reaction in all honesty*,* would be: “great idea*,* love it*,* not this program.*”” (Participant Eight - Group B -Private sector)
	Quote 19: *“Often the timelines preclude anything other than whatever sample you can get pretty quickly… industry pharma clients tend to have tighter timelines because they’re pushing for*,* you know*,* some sort of regulatory communication or some sort of protocol lock… preset long before the PRO developers are engaged.”* (Participant Seven - Group B - Private sector)
**Theme: Socio-political context**	
USA-based participants	Quote 20: “*We’ve gotten better about recruiting from different geographical areas*,* looking at socio-economic status*,* to the best of our ability*,* I don’t think we’re very good at measuring that at all or assessing that*,* but trying to look at different levels of socio-economic status*,* race and ethnicity. To a degree*,* sexual orientation or gender identity… after the past couple months*,* I don’t know… I mean*,* I guess we’re not doing that anymore*.” (Participant Eight - Group B - Private sector)
	Quote 21: *“So at UMass Boston*,* I felt like I got incredible training on… how to be inclusive in research in the questions that we ask. And then I went to a few clinical placements where that was not the case. I felt like I went back in time 15 years*,* which is kind of what the United States feels like right now.”* (Participant Six - Group A – Private sector/Public sector - USA based)
Non-USA-based participants concerns	Quote 22: “*I think we are going in the right direction …. I think we are starting to kind of see*,* OK*,* diversity doesn’t just mean race and ethnicity… [But]with Trump in the US having a big impact on the FDA guidances*,* you know*,* I don’t know if it will keep going in the positive direction it has been…”* (Participant Five - Group B - Private sector)

**Table 4:** Perceived facilitators to using intersectionality to inform fit-for-purpose PROM development – Supporting Illustrative Quotes

Descriptions	Supporting Illustrative Quotes
**Theme: Including different populations (Sub-theme: Community-based participatory research)**	
	Quote 23: “*You’ve got to get really clear constructs that come from the minds of community members or the target population… Rather than doing an item soup and seeing what numbers come up from the soup*.” (Participant One - Group B - Academic/Public sector)
**Theme: Including different populations (Sub-theme: Diversifying research teams)**	
The importance of a diverse socio-demographic research teams	Quote 24: *“We were able to gather a big team with diverse experiences and diverse fields of knowledge*,* and this is definitely one of the things that was key for what*,* I believe*,* was [a] really successful project.”* (Participant Two - Group A - Academic/Public sector)
Diversity in research teams enabled issues with PROMs to be more easily identified	Quote 25: *“If it literally would not be fit-for-purpose within someone’s own home or household*,* then why would it make sense anywhere else?”* (Participant Four - Group B - Academic/Public sector)
Diverse research teams help build trust with communities who see people like them reflected in teams	Quote 26: *“Trust isn’t built institution to institution*,* it’s finding the right people. You know*,* friends of mine who are qualitative researchers and who are black are*,* are*,* like: “you*,* A need me to go in*,* like as a white person*,* you can’t just walk in and be like*,* hey*,* trust me”*,* no matter what your motives are.”* (Participant Eight - Group B - Private sector)
The importance of multidisciplinary research teams	Quote 27: *“We also had like a patient advisor. She’s a person that is from patient led collaborative research and this was definitely very helpful for us to have this broader understanding and like [patient] centring fashion since the beginning in our research.”* Quote 28: *“Having some kind of intersectionality partner or someone who could be advisor to the team*,* and making a way to*,* to some way to include this patient experience*,* this experiential knowledge.”* (Participant Two - Group A - Academic/Public sector)
	Quote 29: *“We have worked with kind of patient advocates before and… I think that does help… people think of things in a different way… look at things in a bit more detail… [and] make sure we’re challenging everything.”* (Participant Five - Group B - Private sector)
**Theme: Spearheads instigating change**	
Evidence of a spearhead instigating change within the team	Quote 30: *“Even though this one research team member kind of really spearheaded it… and made it kind of a priority*,* no one else in the research team would like*,* they*,* they went with the idea but they didn’t understand like the kind of nuts and bolts of bolts of it.”* (Participant Four - Group B - Academic/Public sector)
PROM researchers are responsible for acting as spearheads and being more reflexive	Quote 31: *“[PROM developers should be] going through that thought exercise of*,* are there ways in which different types of people might experience this differently? And if so*,* make sure we’re writing our questions broadly enough to capture all of those ways in which they experience.”* (Participant Seven - Group B - Private sector)
	Quote 32: *“What more can we do in this position of PRO developers to*,* you know*,* make sure we are asking the right questions and recruiting diverse samples.”* Quote 33: *“I think the researchers themselves as well and kind of acknowledging our own unconscious biases and like what we’re bringing unconsciously into when I’m evaluating PROs …”* (Participant Five - Group B - Private sector)
**Theme: Public body support**	
	Quote 34: *“It’s really the larger sort of more public facing or public funded projects*,* and the consortium driven efforts that are really more focused on it [rigorous and inclusive research methods].”* (Participant Seven - Group B - Private sector)
**Theme: Angles of Persuasion (Sub-theme: Appealing to people’s rigour and selling the problem)**	
	Quote 35: *“I really do think that because people are so different and people come from such different backgrounds that you can’t appeal on the basis of like fairness and equity. You have to do it from a… methodologically robustness point of view. And so… the way that I have done it in my*,* in my work generally is to appeal to people’s rigour. And to appeal to their like research quality*,* and ultimately if you’re developing a PROM…you want it to be fit-for-purpose.”* (Participant Four - Group B - Academic/Public sector)
	Quote 36: *“I don’t think that the problem has been well empirically visualised… we haven’t really showed that*,* you know what*,* people are actually really being harmed… this is not a theoretical issue.”* (Participant Three - Group A - Academic/Public sector)
**Theme: Angles of Persuasion (Sub-theme: Being pragmatic)**	
	Quote 37: *“A good questionnaire can actually not be a burden. It can be the opposite … It can improve recruitment and retention at trial. And I was setting the long HLQ questionnaire with migrants in Portugal. Our team*,* they thought …. “Ah*,* it’s long questionnaire. We might only get 100 or 200 questionnaire results”. I got 1200 responses because the questions were relevant.”* (Participant One - Group B - Academic/Public sector)
	Quote 38: *“We also did an extensive literature review on burden and have really found that the length of the survey is not the determining factor for people. People will answer questions if they think it’s relevant*,* they get upset if there are questions that don’t make sense to their situation.”* (Participant Three - Group A - Academic/Public sector)

### Perceived barriers to adopting intersectional approaches

#### Understanding: “It’s a skill and expertise in its own right”

While most participants indicated they had come across ideas related to intersectionality and demonstrated a high-level understanding, there were some differences in understandings between those in Groups A and B. For example, while Group A participants demonstrated a more comprehensive grasp of intersectionality (Table 3: Q1), most Group B participants explained that they had only come across intersectionality in a general sense without having explored it in-depth (Table 3: Q2-3). Several participants in both groups also noted a lack of understanding about intersectionality in the wider health outcomes field (Table 3: Q4), with one suggesting that this understanding required the development of specific skills:

*You can’t expect everyone to feel the same way nor have the same degree of familiarity with this stuff [intersectionality] because…it is a skill in its own right*,* it’s an expertise and a set of experience in its own right.* (Participant Four - Group B - Academic/Public sector)

Another participant suggested that this lack of understanding might be linked to academic background as, in their experience, team members from a social science background could more easily articulate ideas relating to intersectionality and adopt an intersectional perspective (Table 3: Q5). The importance of academic background was echoed by other participants who described how they had first learned about intersectionality when studying for a social science degree at university (Table 3: Q6).

#### Methodologically challenging

Another common barrier discussed by participants in both groups was not knowing *how* to embed an intersectional approach within PROM development (Table 3: Q7-8), with some noting how translating intersectional thought into practice could be an even more challenging step to take than understanding it:

*[It is one thing to] hear and think it’s [intersectionality] important and the other thing is like how can I successfully use that to inform my research design and conducting the research and also… analysing my findings and communicating that?* (Participant Two - Group A - Academic/Public sector)

#### Methodological rigour

Most participants across both groups and sectors reported a general improvement overall in methodological rigour in PROM development over the past 20 years, particularly since the 2009 release of FDA guidelines on PROM development and validation [2]. Nevertheless, several raised concerns about cases of inadequate methodological rigour amongst some researchers developing PROMs and described this as an ongoing challenge across sectors (Table 3: Q9). They suggested that if existing issues in PROM development persisted, such as understanding what validity means in relation to PROMs or the importance of epistemological transparency, encouraging researchers to take a step further and integrate intersectional thought into PROM development might be challenging.

For example, one participant talked about the “cottage industries of academics” who develop new PROMs and claim them to be “valid” (Table 3: Q10). This concern over PROM validity was also raised by another participant who spoke about the need for a “shift” within the health outcomes field in how validity is understood when evaluating PROMs (Table 3: Q11). Concerns about methodological rigour when developing PROMs were also raised in relation to epistemology. One participant highlighted that, whilst seldom seen in the PROM sector, it is common for qualitative researchers in other disciplines to outline their epistemological stance (Table 3: Q12).

Insufficient methodological rigour when developing PROMs was therefore perceived as a barrier because, as participant eight argued, researchers and industry must first recognise shortcomings with existing methods and practices before meaningful change can occur and intersectional thought can be embedded into PROM development:

*[If] everyone feels like the science is good enough already*,* I don’t think in industry you’re going to find enough of a push to say*,* “let’s step back and do it more rigorously.”* (Participant Eight - Group B - Private sector)

#### Hard-to-reach groups

Participants in both groups stressed the importance of involving diverse individuals in PROM development; for example, in concept elicitation and cognitive debriefing interviews. However, several, notably from Group B, suggested that a considerable barrier arose from the unwillingness of underserved and/or more diverse populations to participate in PROM development research and/or difficulty reaching them (Table 3: Q13-14). Such participants cited mistrust of the medical community, arising, for instance, from the influence of historical abuses such as the Tuskegee Syphilis Study in America (Table 3: Q15) or medical abuses experienced by members of the trans community:

*…trans people especially at the moment… [have been] horrifically…marginalised and*,* you know*,* have had horrible experiences with healthcare providers. So…why would they then…help us out by taking part in this research.* (Participant Five - Group B - Private sector)

#### Financial Barriers

Financial barriers to adopting more intersectional approaches were a central thread running through many accounts; this included vested financial interests and tight timelines and budgets.

#### “McDonaldisation of PROs”

The “McDonaldisation of PROs” was an expression coined by one participant to describe the vested financial interests and power structures entangled in PROM development and use which, they suggested, can create resistance to change (Table 3: Q16). The reference to McDonald’s was intended to highlight the scale of these financial interests, with the participant noting that “it’s a very big industry to keep the status quo”. This sentiment was shared by another participant who described how the field has “invested huge amounts” into the development of existing PROMs leading to a tension between the continued use of established, profitable PROMs and the push for changes in PROM development practices:

*Once you open the equity and diversity bucket*,* it questions the validity of all these standardised tools and that*,* that is of course also very much a threat to the field. So*,* I think there is a tension there. People have invested huge amounts…into the developments of these tools.* (Participant Three - Group A - Academic/Public sector)

*Once you open the equity and diversity bucket*,* it questions the validity of all these standardised tools and that*,* that is of course also very much a threat to the field. So*,* I think there is a tension there. People have invested huge amounts…into the developments of these tools.* (Participant Three - Group A - Academic/Public sector)

#### Timelines and budgets

Whilst none of the participants working in the private sector reported having previously published intersectionality-related PROM research, many discussed their attempts to incorporate more inclusive approaches into PROM development and described experiencing challenges with tight budgets and study timelines. This was acutely felt by the participants who were working with pharmaceutical companies. They described how the for-profit nature of the pharmaceutical industry meant that PROM science must always contend with commercial priorities which limited the feasibility of rigorous and intersectional approaches to PROM development (Table 3: Q17-18), with one participant recounting how:

*I never worked at the University of Lilly. I worked for Eli Lilly and Company…it’s science based*,* but it’s not science only.* (Participant Eight - Group B - Private sector)

For some, timeline pressures were considered an even greater barrier. For example, participant seven described how deadlines could be set before conversations with PROM developers were even initiated (Table 3: Q19). It was further noted that timeline pressures, along with budgetary constraints, could restrict sample diversity in PROM development studies because more inclusive approaches, such as community-based participatory research (CBPR), took longer to initiate, sustain and cost more money.

#### Socio-political context

Broader socio-political changes were raised by participants from both groups as well as across sectors and geographic locations. Largely, discussion of these socio-political changes focused on those happening in the USA following US President Trump’s inauguration in January 2025. These included systematic cutbacks to diversity, equity, inclusion (DEI) related policies [35], as well as considerable cuts in resources for public-health projects with the FDA witnessing 20,000 job cuts [36]. DEI-related guidance was also removed from the FDA website including “Diversity Action Plans to Improve Enrollment of Participants from Underrepresented Populations in Clinical Studies” [37]. This particular guidance document outlined how to enrol and retain representative clinical-trial participants, especially from underserved communities, to ensure that all clinically relevant populations are included in studies. Against this backdrop, many participants related challenges with intersectional PROM development to the current socio-political context, especially those who were USA-based and who expressed despondency about the future of more inclusive PROM research (Table 3: Q20-21).

However, participants outside of the USA also discussed how these events impacted them. One discussed how this socio-political context could potentially hinder the use (in the USA) of a new DEI-related PROM they might be developing. Other participants noted how the removal of some FDA guidance and initiatives from the FDA website had affected their daily work following US President Trump’s executive order to eradicate DEI-related information from government sources (Table 3: Q22) with participant four describing how:

*It’s such a shame because it’s really going to slant how people see things…we know that NIH [National Institutes of Health] like grants have been kind of paused*,* and like they’re literally just doing like a search*,* like a word search for like anything that has like women*,* or diverse*,* or like race*,* or things like that… I know from my own work*,* that like several of the initiatives that I would often refer to have disappeared from the Internet*. (Participant Four - Group B - Academic/Public sector)

### Perceived facilitators to using intersectionality

Participants proposed a range of solutions, with Group A largely drawing on their own experience of engaging with intersectionality during PROM development, while Group B participants referred to their individual attempts to develop more inclusive PROMs.

#### Including different populations

Two sub-themes were identified: encouraging more diverse research samples to be involved in PROM development through CBPR and increasing research team diversity.

#### Community-based participatory research (CBPR)

Ideas related to CBPR were raised by several participants to more successfully engage with hard-to-reach groups. Participants suggested that CBPR would elicit a broader range of perspectives which better reflect the experiences of the target population (Table 4: Q23) because:

*The more we get sort of your typical community-based provider comfortable with research*,* and then being able to partner there and get patients who aren’t going to the ‘University of Perfection’ for treatment… I think you get already a more diverse population.* (Participant Eight - Group B - Private sector)

Participant five also argued that by giving participants “more of an active involvement… [to] guide the research…”, researchers can build trust by showing participants how “it’s in their interest to participate as well”, which, they reasoned, would improve overall “interest and… engagement”.

#### Diversifying research teams

Most Group A participants shared experiences of how working in more diverse research teams facilitated more intersectional approaches (Table 4: Q24) because:

*[In my experience] when there are people of colour*,* or folks with marginalised identities…on the team*,* the more there were*,* the less convincing was needed.* (Participant Six - Group A - Private sector/Public sector)

Other participants from Group B also emphasised the value of diverse research teams, arguing that this diversity enabled potential issues with PROMs to be more easily identified because the researchers could often recognise when aspects of a PROM might or might not be suitable for someone in their own community (Table 4: Q25). Another proposed advantage of working in diverse research teams was that it would build “trust” with research participants through an improved understanding of communities and by communities feeling better represented within research (Table 4: Q26).

Most participants, in both groups, referred to socio-demographic diversity while a few also referred to multidisciplinary diversity for encouraging more “diverse opinions and methods”. Other participants underlined the importance of collaborating with external partners; for instance, by involving an intersectionality advisor and/or patient advocate (Table 4: Q27-29).

#### “Spearheads” instigating change

Several participants in both groups also reflected on the need for an individual to instigate and drive change. One participant (Group B) used the term “spearhead” to depict a colleague who had played a pivotal role in encouraging a more inclusive PROM research approach (Table 4: Q30). Another participant (Group A) also underlined the significance of certain individuals in their team who they described as “conductors” for “changing scientific practices, changing researchers’ practices”. Such accounts echoed the suggestions from other participants that PROM researchers themselves are responsible for driving a shift in research practices (Table 4: Q31). Such participants suggested, for example, that PROM researchers should be more reflexive about how their positionality and unconscious biases might impact their research (Table 4: Q32-33).

#### Public body support

The value of public body support was also made clear by most participants, notably in relation to funding. For example, one participant discussed how their publicly funded grant for equitable people-centred health measurement:

*… ranked number one in the country. So*,* it did actually resonate in the end*,* but it took a lot to get there*. (Participant Three - Group A - Academic/Public sector)

Group B participants also stressed the significance of public body support, with one noting that from their USA perspective, more inclusive and rigorous study designs were largely those with public body support (Table 4: Q34). Another participant likewise remarked that a key enabling factor for their equitable PROM research in the UK was funding from their local research network:

*…it happened at the time that there was…funding available from our local research network that was about increasing diversity… And I mean since then*,* given…the mainstream attention that’s been directed at…inclusive research*,* there has been more money forthcoming.* (Participant Four - Group B - Academic/Public sector)

#### Angles of persuasion

When asked about *how* to encourage a shift in approaches to PROM development, participants proposed ideas which fell into two sub-themes: appealing to people’s rigour and being pragmatic.

#### Appealing to people’s rigour and selling the problem

Several suggested that appealing to researchers’ desire to conduct rigorous science could be used to persuade them to adopt more intersectional PROM development practices. For example, one participant outlined how PROM developers seek to construct measures that *are* genuinely fit-for-purpose. As such, they explained how emphasising the importance of methodological rigour to develop PROMs which are genuinely fit-for-purpose could be an effective angle of persuasion (Table 4: Q35). However, participants suggested that before appealing to researchers’ commitment to rigour, effort would be required to “sell the problem”; namely, the consequences for patient health of using PROMs which are poorly developed. For this reason, participants suggested that evidence-based arguments outlining the impact and harm to health of overlooking intersectionality were key to persuading researchers that it was important to adopt intersectional approaches to develop fit-for-purpose PROMs (Table 4: Q36).

#### Being pragmatic

To tackle some of the financial barriers, such as the McDonaldisation of PROs along with tight timelines and budgets, participants emphasised pragmatic angles of persuasion. Several argued that the financial soundness of changing practices should be stressed by highlighting the economic implications of *not* developing a PROM that is fit-for-purpose:

*If you’re developing something*,* you want it to be fit-for-purpose. Now if… it will not be fit-for-purpose for that group. Then it’s just a complete waste of time and money.* (Participant Four - Group B - Academic/Public sector)

Other participants also discussed how developing fit-for-purpose PROMs is a financially sound investment that can encourage greater research participant retention as well as better PROM response rates since people feel that the questions are more relevant to them (Table 4: Q37-38).

## Discussion

### Summary of findings

The relative absence of intersectionality within health outcomes research [38] prompted this exploratory qualitative study to understand and explore PROM researchers’ views about the barriers and facilitators to integrating intersectional thought within PROM development.

Study participants highlighted important barriers, including, limited understanding of intersectionality, as well as shortcomings in methodological rigour during PROM development, both of which were aggravated by the socio-political context and challenges engaging with diverse populations. Nevertheless, participants stressed the feasibility of adopting more intersectional approaches by engaging with more representative populations and research teams, appealing to PROM developers’ desire for methodological rigour and selling the problem, as well as being pragmatic.

### Comparison with existing literature

Mirroring studies undertaken by other intersectionality scholars [39–43], participants reported limited understanding of intersectionality, its value for health, and uncertainty about how to apply an intersectional lens. As they noted, while PROM development methods have become more rigorous over the past two decades [44], with the FDA’s PFDD guidelines indicating progress [11], concerns persist, particularly regarding validity and its measurement. These concerns mirror those raised by others [4, 45], notably, Bauer, an intersectionality and population health scholar, who argued that “the importance of intersectionality may be better grasped by researchers if its relationship to core methodological (e.g. validity) concerns were made clear” [46]. Additionally, whilst the need for greater epistemological transparency was voiced by some participants, this concern has also been raised by intersectionality scholars, including Abrams who noted that “methodology based on intersectionality is incomplete without epistemology” [47].

Challenges recruiting diverse samples were highlighted as a major barrier, consistent with studies exploring PROM developers’ views about including patients in PROM development [48] and the wider health research literature [49–50]. In line with our findings, a key reason appears to be mistrust of healthcare professionals and researchers [51].

The high cost of conducting health research is well known [52–53], and efforts to introduce more equitable research practices have faced financial resistance [54]. Participants’ concerns regarding the pharmaceutical industry placing pressures on researchers to keep costs low aligns with this broader context of financial restraint. Moreover, numerous researchers have highlighted the need for better public funding to facilitate more equitable health research [55–56].

Although unanticipated at the outset, the socio-political context featured prominently, likely because data collection coincided with the first 100 days of US President Trump being in office and very significant health policy changes. However, the influence of socio-political context on health outcomes research has been documented elsewhere, especially in moments of high political tension [57]. This underscores the value of considering contextual influences, such as socio-political shifts, and their implications for policy, funding priorities, and regulatory uncertainty among health outcomes stakeholders working to change research practices in the future.

Participants’ suggestions for how to adopt more intersectional approaches resonate with the broader health and intersectionality literature. Notably, there have been longstanding calls for more representative research populations [58–60], with the importance of forming diverse research teams also being endorsed in the health literature [58, 61]. Beyond socio-demographic diversity, intersectionality scholars have stressed the value of multidisciplinary diversity and collaborating with external advisors [61], such as intersectionality partners and patient advocates.

Several participants emphasised researchers’ role in spearheading intersectional approaches, echoing wider debates about accountability for equitable health research practices, with Castillo describing how “researchers have the responsibility to bridge divides between scientific innovation and societal impact” [62]. However, scholars exploring intersectionality and health also note that adopting an intersectional lens is not straightforward, and requires self-reflection and the questioning of current practices [41, 61]. Participants’ discussion of reflexive practice thus aligns with intersectionality scholars [41, 47, 63] and qualitative researchers alike [64–66] who emphasise the importance of reflexivity to achieve rigorous research.

Appealing to researchers’ desire to conduct rigorous science and selling the problem of using PROMs developed without considering intersectionality were proposed as an effective angle of persuasion to encourage research practice changes. This aligns with prior work from health outcomes academics showing that PROM development conducted with homogeneous populations increases the risk that PROM results do not accurately reflect the health outcomes they purportedly measure [14]. Part of selling the problem also encompasses highlighting the positive practical implications of considering intersectionality, with other health researchers also linking patient engagement to improved study enrolment rates, funding, and pertinent outcome selection [67].

Many of our findings have parallels with the wider literature, however, rather than considering them in silos, this study has taken an initial step towards showing how an intersectional lens can help bring these various barriers and facilitators together to develop PROMs which are more fit-for-purpose.

### Strengths and Limitations

To our knowledge, this is the first study to investigate intersectionality in relation to PROM development. However, as our study was exploratory, our sample size was necessarily small, and we only interviewed three participants who had previously engaged with intersectional thought, limiting our ability to draw definitive conclusions. Moreover, our sample largely comprised researchers working in high-income countries. To protect their identities, we have not reported key demographic information (e.g., gender and ethnicity), precluding us from considering the potential differences in experiences and accounts based on demographic factors.

Nevertheless, we strove to explore a range of expert experiences across different settings, geographies, and disciplinary backgrounds which, combined with placing the findings in the context of the wider literature, allows readers to cautiously consider our findings’ transferability to their own settings. We also believe that the reflexive approach used throughout this study helped sensitize us to the influence that participant differences had on data collection and interpretation. Participants, however, were not masked from the interviewer’s role as a COA specialist, and this may have influenced the type of information they chose to share. Future studies should consult a larger group of participants, particularly those meeting Group A criteria and from lower to middle income countries, to examine how these findings compare across sectors, geographical regions, and with types of PROMs (for example generic vs. disease-specific).

### Recommendations

Our findings feed into two types of potential recommendations. The first centres on facilitating a culture shift in PROM development practices to reflect intersectional thought, to ensure that PROMs are more fit-for-purpose for the heterogenous populations they are designed to serve. These recommendations include: selling the problem of poor PRO measurement to researchers and decision-makers, improving PROM researchers’ understanding of intersectionality, developing clearer guidelines to assist researchers in adopting intersectional approaches to PROM development, and researchers standing up to spearhead and drive PROM development changes. The second set focuses on ways to develop PROMs through a more intersectional lens by: researchers being transparent about their epistemological stance and more reflexive throughout the whole research process, improving engagement with more diverse research populations through CBPR, establishing more diverse research teams, and being flexible with research designs as a one-size-fits-all approach does not allow for meaningful intersectional PROM development. A detailed description of these recommendations, alongside some suggested resources, is provided in Table 5.

**Table 5:** Summary of recommendations

Objective	Recommendation	Examples and details
Facilitate a shift in culture towards more intersectional approaches to PROM development	Sell the problem	Appeal to researchers’ desire to conduct rigorous science, e.g. more studies on the impact of poor health outcome measurement and problems with the validity of many PROMs. More evidence-based work is needed to spark consensus in the field that change is necessary.
		Be pragmatic, e.g. fit-for-purpose PROMs can result in more financially sound choices, better study recruitment and retention, and improved decision-making.
	Improve understanding of intersectionality	Disseminate resources within the field. Build on existing resources and adapt to the health outcomes sector. *Example Resources*: • United Nations *Intersectionality Resource Guide and Toolkit* [25] • Sabik’s *Intersectionality Toolbox* [68] • Abrams’ *Considerations for Employing Intersectionality in Qualitative Health Research* [47]
		When working with external recruitment vendors, implement intersectionality and DEI training to support inclusive recruitment practices.
	Clearer guidance	Establish clearer guidance outlining what intersectionality is, its value for PRO measurement, and how to implement these ideas in practice. Societies such as the International Society for Quality of Life Research (ISOQOL) are well-placed to develop such guidance considering its influence in the sector and commitment to supporting the patient voice in patient-reported outcomes research [69].
	Be a spearhead - instigate change	Change will necessitate a shift in funding but also fellow researchers to support spearheads. Such action should include individuals pushing the boundaries of standard study designs as well as public bodies financially supporting intersectional research. Instigating change may not always be rewarded by funding opportunities, yet fellow researchers can recognise when colleagues are pushing for change and support initiatives however they can.
Recommendations for how to approach PROM development through a more intersectional lens	Transparent epistemological stance	By considering “epistemological moorings” [70], researchers would have a clearer sense of how the knowledge they generate connects to broader systems of oppression and the role of intersectional populations in knowledge production. This push for transparency could also come from journal requirements.
	Reflexive practice	Understand what reflexivity is and how to engage with it meaningfully from the conceptualisation of the research process through to implementation. *Example Resource*: • United Nation *Intersectionality Resource Guide and Toolkit* [25] – see discussion of reflexivity as an ‘enabler’
		Appealing to researchers’ methodological rigour would be important for encouraging reflexivity as would journals requiring reflexivity in PROM development publications. Several qualitative research journals in the social sciences already have such requirements [71] and so similar expectations could be applied to health outcomes research journals.
	Engage with diverse research populations	Make use of existing guidance in a meaningful way. *Example Resources*: • *REP-EQUITY toolkit* [72]
		Engage with more community-based participatory research methods. *Example Resources*: • CBPR guidance in Table 1 [73] • Guideline 7 of from the Council for International Organizations of Medical Sciences [74]
	Establish diverse research teams	Make use of existing guidance in a meaningful way. *Example Resources*: • Best practices in equity, diversity and inclusion in research practice and design [75]
		Foster both socio-demographic diversity as well as multidisciplinary diversity.
		Collaborate with external partners (e.g. intersectionality partner and/or patient advocates) to support the integration of intersectional perspectives and facilitate a patient-centric approach.
	Be flexible: One size does not fit all	Move away from checklists when developing PROMs as they do not foster in-depth reflection about the most well-adapted methods [76]. An intersectional approach to PROM development should be centred on adapting research to ensure the PROM’s intended context and population, both of which are key tenants of intersectionality [47], remain the focal points.

## Conclusion

This exploratory study has made a necessary and meaningful first step towards better understanding researchers’ views on using intersectional approaches to develop fit-for-purpose PROMs. The findings offer some insight into the potential value of intersectionality as a framework for addressing existing PROM shortcomings along with highlighting several key challenges to doing so. We have also provided tentative recommendations for overcoming such barriers and adopting more intersectional approaches to PROM development. We hope that these recommendations will stimulate further research and encourage the development of PROMs which are more fit-for-purpose for heterogeneous populations, and that ultimately outcomes which are more meaningful for patients are considered in health-care decision-making.

## Supplementary Information

Below is the link to the electronic supplementary material.

Supplementary Material 1

## Data Availability

The complete dataset generated and analysed during the current study are not publicly available due to reasons of sensitivity and privacy to protect the participants. Extracts from the dataset supporting the conclusions of this article are included within the article. Some additional data extracts may be made available from the corresponding author upon reasonable request.

## Abbreviations

CBPR: Community-based participatory research
COA: Clinical Outcome Assessment
FDA: Food and Drug Administration
NIH: National Institutes of Health
PROM: Patient-Reported Outcome Measure
QoL: Quality of Life
