# Supplementary material for: Using intersectionality to inform fit-for-purpose patient-reported outcome measure development: a qualitative interview study
Source: J Patient Rep Outcomes. 2026 Jul 24;10:169. doi: 10.1186/s41687-026-01132-y (PMC13612776; doi:10.1186/s41687-026-01132-y)
Supplement: Supplementary file 1 [file 41687_2026_1132_MOESM1_ESM.docx]

**Additional Materials:** *Using* *intersectionality to inform fit-for-purpose patient-reported outcome measure development: A qualitative interview study*

Appendix A: Literature review results (p. 2)

Appendix B: COREQ Checklist (pp. 3-7)

Appendix C: Topic guide A (pp. 8-12)

Appendix D: Topic guide B (pp. 13-17)

References (pp. 18-21)

Appendix A

Table A1: Results of the PubMed Review into PROM Development and Intersectionality

| **Acronym** | **Full Name** | **Year/Reference** |
| --- | --- | --- |
| **Measures Developed with a Consideration of Intersectionality** | | |
| LGBT-POC | The LGBT People of Color Microaggressions Scale | 2011 [1] |
| GRMSAAW | Gendered Racial Microaggressions Scale for Asian American Women | 2018 [2] |
| GRSAM | Gendered Racism Scales for Asian American Men | 2018 [3] |
| SMASI | Sexual Minority Adolescent Stress Inventory | 2018 [4] |
| ESWS | Experiences of Sex Work Stigma | 2021[5] |
| MIDAS | Multidimensional Inventory of Deaf Acculturative Stress | 2022 [6] |
| N/A | Intersectional Stigma Scale | 2023 [7] |
| G-SISI | Gay-Specific Intraminority Stigma Inventory | 2023 [8] |
| N/A | Gender-Diversity and Autism Questionnaire | 2023 [9] |
| RIASS | Reproductive Injustice Across Systems Scale | 2024 [10] |
| N/A | Long Covid Survey (no official name provided) | 2024 [11] |
| **Retrospective Intersectional Interpretation of a PROM** | | |
| EDE-Q | Eating Disorder Examination-Questionnaire | 2022 [12] / 2024 [13] |
| PHQ-8 | 8-item patient health questionnaire | 2023 [14] |
| EES-R | Emotional Eating Scale-Revised | 2024 [13] |
| PMQ | 5-item Parental Monitoring Questionnaire | 2025 [15] |
| **Tools to Measure Intersectionality to be Used Alongside Other PROMs** | | |
| BMES | Black Men's Experiences Scale | 2016 [16] |
| IDI | Intersectional Discrimination Index | 2019 [17] |
| N/A | Marginalized Identity Score | 2020 [18] |
| SAFE | Socially Ascribed intersectional identities For Equity | 2024 [19] |
| N/A | Jeopardy Index | 2025 [20] |
| Literature review conducted on intersectionality and PROM development in August 2024 which retrieved 614 results and was supplemented by forward and backward searches as well as grey literature searches. Further targeted literature searches were conducted throughout the study until May 2025. | | |

Appendix B

Table B1: Consolidated Criteria for Reporting Qualitative Studies (COREQ): An Adapted 32-item Checklist [21]

| **Item** | **Guide questions/descriptions** | **Additional Details** | **Page number where discussed in publication** |
| --- | --- | --- | --- |
| **Research team and reflexivity** | | | |
| 1. Interviewer | Which author/s conducted the interviews? | Tilly Stott | pp. 6, 8-9 |
| 2. Credentials | What were the researcher’s credentials? | Master of Public Health (MPH) | p. 6 |
| 3. Occupation | What was their occupation at the time of the study? | Research Associate (Clinical Outcome Assessment (COA) specialist) | p. 6 |
| 4. Gender | Was the researcher male or female? | Female | p. 6 |
| 5. Experience and training | What experience or training did the researcher have? | The interviewer had undergone qualitative interviewing training and had 5 years of experience working with COAs (mainly PROMs) | p. 6 |
| 6. Relationship established with participants | Was a relationship established with participants prior to study commencement? | No relationship was established prior to study commencement between the interviewer and interviewees. | N/A |
| 7. Participant knowledge of the interviewer | What did the participants know about the researcher? *e.g. personal goals, reasons for doing the research* | As part of the informed consent process, all potential participants were emailed a detailed participant information form which outlined the study background, objectives, and what would be expected from the participants. | p. 8 |
| 8. Interviewer characteristics | What characteristics were reported about the interviewer? *e.g. bias, assumptions, reasons and interests in the research topic* | The relevance and importance of the topic were detailed in the participant information form to provide background to the researchers’ interest in the topic. | N/A |
| **Study Design** | | | |
| 9. Methodological orientation and Theory | What methodological orientation was stated to underpin the study? | This study and its design drew on key tenets of grounded theory (GT). GT emphasises a data-driven research process [22] which was critical since there is very little research on the role of intersectionality in PROM development. Basing the findings on PROM researchers’ direct experience and viewpoints was therefore essential [23-25]. A particular influence was Charmaz’s constructivist GT stance [26]. We believed that data analysis is a construction based on our interpretation of what interviewees share which is aligned with Charmaz’s thoughts on the relationship between one’s positionality and role as a researcher [24] | p. 6 |
| 10. Sampling | How were participants selected? | Potential participants were identified in the literature because explore a specific topic in-depth and to target participants with specific experiences.  To supplement our recruitment, we asked potential participants to recommend fellow researchers who they thought fitted well with our inclusion criteria. This was because there is limited research related to intersectional PROM development and so tapping into the networks of researchers who had responded to our initial email was an opportunity to identify thought leaders [27]. | pp. 6-8 |
| 11. Method of approach | How were participants approached? | Participants were contacted by email since they were based worldwide, and the majority had a public webpage and/or publications with email contact details. | p. 8 |
| 12. Sample size | How many participants were in the study? | 8 | p. 10 |
| 13. Non-participation | How many people refused to participate or dropped out? Reasons? | 24 potential participants were contacted who did not partake in the study. Some did not provide a reason but reasons for refusals were primarily related to the narrow data collection window (Jan-March 2025). However, several researchers also implied that the socio-political situation in the USA meant that they were reluctant to partake. | p. 8 |
| 14. Setting of data collection | Where was the data collected? | Data was collected via a Microsoft Teams video-call. The interviewer conducted the calls at home, and the interviewees were either at home or in their place of work. | p. 8 |
| 15. Presence of non-participants | Was anyone else present besides the participants and researchers? | No | N/A |
| 16. Description of sample | What are the important characteristics of the sample? | Participants (n=8) worked on PROMs used in a variety of contexts including clinical trials, hospital services, government programs, and clinical practice. They were based in the USA, UK, Canada, Brazil, and Australia/France. Three participants were recruited who had explicitly engaged with intersectionality in published PROM research literature and 5 who had not.  To protect participants’ anonymity, a high-level overview of interviewees’ background is presented in Table 2. | p. 10 |
| 17. Interview guide | Were questions, prompts, guides provided by the authors? Was it pilot tested? | A topic guide was used by the interviewer to provide a rough outline to the semi-structured interviews and was updated iteratively as more interviews were conducted and new ideas generated. The preliminary topic guide was pilot-tested with two external PROM experts who were not involved in the study. The final topic guides (one per group) are available in appendix C and D. | pp. 8 |
| 18. Repeat interviews | Were repeat interviews carried out? If yes, how many? | No | N/A |
| 19. Audio/visual recording | Did the research use audio or visual recording to collect the data? | The audio, video, and transcripts of the interview were recorded using Microsoft Teams. | p. 8-9 |
| 20. Field notes | Were field notes made during and/or after the interview? | Field notes were made after each interview to support the contextualisation of transcripts during analysis.  Regular journalling was also practiced before and after interviews as part of the reflexive process to help “bracket” the interviewer’s experience and emotive reactions [28]. | N/A |
| 21. Duration | What was the duration of the interviews? | Interviews ranged between 53 minutes and 1 hour 9 minutes | p. 8 |
| 22. Data saturation | Was data saturation discussed? | Saturation was not used as a concept to define the sample size as we would have been unlikely to attain saturation within the three-month recruitment and data collection timeframe. Furthermore, there is considerable controversy about what can be meaningfully understood when researchers claim to ‘reach saturation’ [29-30]. As such, the intention was to collect data of adequate depth and breadth to provide meaningful answers to the research questions [30]. | N/A |
| 23. Transcripts returned | Were transcripts returned to participants for comment and/or correction? | All participants were provided with the opportunity to comment on and make changes to the transcripts. | N/A |
| **Analysis and findings** | | | |
| 24. Number of data coders | How many data coders coded the data? | 2 | p. 9 |
| 25. Description of the coding tree | Did authors provide a description of the coding tree? | Yes | p. 9 |
| 26. Derivation of themes | Were themes identified in advance or derived from the data? | Themes were not identified in advance, they were derived bottom-up (inductively) from the data. Although umbrella themes (facilitators and barriers) were pre-defined based on our research question. | p. 9 |
| 27. Software | What software, if applicable, was used to manage the data? | Nvivo14 [32] | p. 9 |
| 28. Participant checking | Did participants provide feedback on the findings? | No | N/A |
| 29. Quotations presented | Were participant quotations presented to illustrate the themes / findings? Was each quotation identified? | Yes, see the Results, Table 3 and Table 4 | N/A |
| 30. Data and findings consistent | Was there consistency between the data presented and the findings? | Yes, see the Results section. | N/A |
| 31. Clarity of major themes | Were major themes clearly presented in the findings? | Yes, see the Results section. | N/A |
| 32. Clarity of minor themes | Is there a description of diverse cases or discussion of minor themes? | To some degree. For example, only a couple of participants discussed *being pragmatic* as an effective angle of persuasion. The same applies to the *McDonaldisation of PROs*. However, for the sake of pertinence, clarity, and the word constraints of this publication not all codes and themes have been described in the results. However, it should be noted that the process of developing these codes and themes listed above informed the final results presented. | N/A |

Appendix C:

Topic Guide A: Group A (for researchers who have engaged with intersectional thought)

*This interview will be semi-structured, the order of the questions below may vary for each interview as well as the content if new themes are raised by participants. Equally, not all questions listed below will be asked and will be adapted to participant content as informed by Charmaz [24].*

**Rapport** – e.g. where are they based, what’s the weather like etc.?

**Introduction**: Thank you for volunteering to take part in this study. Today I will be asking you questions about your experience of developing PROM(s), with a focus on what makes PROMs fit-for-purpose and how this may or may not relate to intersectionality. There are no correct answers, the main objective is to explore your experience and hear about your perspective on PROM development. If you wish to stop the interview and/or withdraw from the study you may do so at any point.

**Consent**: Before we get started, I just want to confirm that as stated on the consent form that you still agree to me recording this interview and that you understand that what you will say will be used and may be quoted in the study?

[If they opted in for not being anonymous] I would also like to confirm that as you opted in on the consent form that you are still happy not to be anonymous?

**Misc.**: I also wanted to say that I will be referring to the topic guide for today as we go through the interview, so if you see me looking at my other screen I am just checking back at my guide. I will also be taking written notes if you notice me looking down.

Do you have any questions before I start the interview?

**Recording**: I will now start the recording and we can get things underway.

| **Topic** | **Sub-topic** | **Questions** | **Probing** |
| --- | --- | --- | --- |
| Introduction | Professional and/or academic background | 1. Tell me a bit about your professional and/or academic background? |  |
|  | PROM development experience | 2. How do you understand the term patient-reported outcome measure (PROM)? |  |
|  |  | 3. How did you first come to work on PROM development? | - What was your role in this development process? - If you remember, tell me about what the first PROM that you developed measured and in what population it was for? |
|  |  | 4. What is the most recent PROM that you have developed and how were you involved in that process? | - What was this PROM measuring and who was it designed for? - What was the sampling strategy for this PROM, were there any particular populations that you were trying to target? |
|  |  | 5. Could you tell me a bit about how you decide on what steps to follow when developing a PROM? | - Experience, guidelines etc. |
|  |  | 6. Do you think the way in which PROMs are developed has changed over time and if so please outline in what ways. This can either be from what you have seen in your own experiences or with what you see in the PROM literature? |  |
| Intersectionality | Understanding of intersectionality | 7. In your paper XXX, you mentioned intersectionality in relation to XXX, explain to me how you understand the term intersectionality?^[1]^ | - How did you first come across ideas related to intersectionality? - *[If the participant asks what the definition is, provide them with Collins]* I wanted to ask if, and if so how, you feel that this definition resonates with you or not? Is this understanding of intersectionality something that you feel is relevant for PROM development? |
| Barriers & Facilitators | Experience of intersectional PROM development | 8. As we have discussed, in the development paper for the [PROM that the researcher developed], you discussed intersectionality. How did it come about that intersectionality was discussed in this paper? | - What did your team do to embed intersectional principles into the study design of your PROM development? - How would you describe the team’s attitude to adopting a more intersectional approach and what do you think underpinned these views? |
|  | Challenges | 9. Did the team encounter any challenges when developing the [PROM that the researcher developed] and if so what were they? 10. Based on your experience of developing PROMs more generally, have you faced any other challenges when developing PROMs and if so what have they been? | - What were the reasons why you and your team faced these challenges? - How do you think that intersectional thought may have assisted with or hindered these other challenges? |
|  | Awareness of intersectional PROM development | 11. What other PROMs are you aware about that have implicitly or explicitly integrated intersectional thought into their study design? | - Have you noticed any patterns/similarities between these PROMs developed using an intersectional lens, *e.g. population, what they are measuring etc.*? - Have you come across references to intersectionality in the health outcomes literature and if so what has this been in relation to? |
|  | Fit-for-purpose | 12. Had you come across the term ‘fit-for-purpose’ before this interview, and if so please tell me when you did and what you understand this term to mean? |  |
|  |  | 13. What do you think is lacking most in PROMs that are developed today? 14. Are there any aspects of the PROM development process which could make PROMs more fit-for-purpose? | - How would you advice researchers to go about making their PROMs more fit-for-purpose. - Based on this understanding of fit-for-purpose, how do you perceive an understanding of intersectionality to be relevant (or not) to developing a PROM that is fit-for-purpose? |
|  | Benefits | 15. Do you believe that the [PROM that the researcher developed] is fit-for-purpose? If so, what makes it fit-for-purpose? | - How big of a role do you think that the research team’s intersectional lens played in supporting the generation of this evidence? |
|  |  | 16. Would you have done anything differently to make the [PROM that the researcher developed] more fit-for-purpose and if so what? | - What stopped the research team from being able to take these steps? |
|  | Research Team Diversity | 17. How would you describe the research team that worked on the development of the X PROM? 18. How would you describe the types of research teams that you have worked with when developing PROMs in the past? | - Were there any differences that you noticed between the research team that worked on the [intersectional PROM that the researcher developed] and previous PROMs? |
|  | Summary | 19. Are there any other factors that influence the development of fit-for-purpose PROMs that you would like to share? |  |
| Overcoming Barriers | How to overcome? | 20. Now that we have discussed some of the challenges involved in developing fit-for-purpose PROMs and intersectional approaches, how do you think these barriers could be overcome? | - Out of these challenges, is there one that stands out in particular and if so could you outline why you think that is? - What advice would you give to a fellow researcher that would like to develop a PROM using an intersectional lens? |
|  | Other changes | 21. Are there any other changes that you would like to see in the way in which PROMs are developed? |  |
| Closing Remarks |  | 22. Is there anything else related to PROM development and/or intersectionality that you would like to share with me – or that you think I ought to know about to better understand your experience as a PROM researcher? 23. Is there anything in particular that you would like to ask me? |  |
|  | Thank you very much for offering your time today for this interview. Your participation is much appreciated. If you chose to hear more from me about the findings or about reading the transcripts for redaction then I will be in touch in the next few months. If not, then thanks again and all the best! | | |

**Stop the recording**

Appendix D

Topic Guide B: Group B (for researchers who have not engaged with intersectional thought)

*This interview will be semi-structured, the order of the questions below may vary for each interview as well as the content if new themes are raised by participants. Equally, not all questions listed below will be asked and will be adapted to participant content as informed by Charmaz [24].*

**Rapport** – e.g. where are they based, what’s the weather like etc.?

**Introduction**: Thank you for volunteering to take part in this study. Today I will be asking you questions about your experience of developing PROM(s), with a focus on what makes PROMs fit-for-purpose and how this may or may not relate to intersectionality. There are no correct answers, the main objective is to explore your experience and hear about your perspective on PROM development. If you wish to stop the interview and/or withdraw from the study you may do so at any point.

**Consent**: Before we get started, I just want to confirm that as stated on the consent form that you still agree to me recording this interview and that you understand that what you will say will be used and may be quoted in the study?

[If they opted in for not being anonymous] I would also like to confirm that as you opted in on the consent form that you are still happy not to be anonymous?

**Misc.**: I also wanted to say that I will be referring to the topic guide for today as we go through the interview, so if you see me looking at my other screen I am just checking back at my guide. I will also be taking written notes if you notice me looking down.

Do you have any questions before I start the interview?

**Recording**: I will now start the recording and we can get things underway.

| **Topic** | **Sub-topic** | **Questions** | **Probing** |
| --- | --- | --- | --- |
| Introduction | Professional and/or academic background | 1. Can you tell me a bit about your professional and/or academic background? |  |
|  | PROM development experience | 2. How do you understand the term patient-reported outcome measure (PROM)? |  |
|  |  | 3. How did you first come to work on PROM development? | - What was your role in this development process? - If you remember, tell me about what the first PROM that you developed measured and in what population it was for? |
|  |  | 4. What is the most recent PROM that you have developed and how were you involved in that process? | - What was this PROM measuring and who was it designed for? - What was the sampling strategy for this PROM, were there any particular populations that you were trying to target? |
|  |  | 5. Could you tell me a bit about how you decide on what steps to follow when developing a PROM? | - Experience, guidelines etc. |
|  |  | 6. In what ways, or not, do you think the way in which PROMs are developed has changed over time, either in your own experience or with what you see in the PROM literature? |  |
| Intersectionality | Understanding of intersectionality | 7. Before this interview had you come across the term ‘intersectionality’ and if so   how do you understand the term intersectionality?^[2]^ | - How did you first came across ideas related to intersectionality? - [If the participant asks what the definition is, provide them with Collins] I wanted to ask if, and if so how, you feel that this definition resonates with you or not? Is this understanding of intersectionality something that you feel is relevant for PROM development? |
|  | Awareness of intersectional PROM development | 8. What other PROMs are you aware about that have implicitly or explicitly integrated intersectional thought into their study design? | - Have you noticed any patterns between the PROMs that have been developed using an intersectional lens, and if so please outline what these are? - Have you come across references to intersectionality in the health outcomes literature and if so what has this been in relation to? |
| Barriers & Facilitators | Fit-for-purpose | 9. Had you come across the term ‘fit-for-purpose’ before this interview, and if so please tell me when you did and what you understand this term to mean? | - Based on this understanding of fit-for-purpose, how do you perceive an understanding of intersectionality to be relevant (or not) to developing a PROM that is fit-for-purpose? |
|  |  | 10. What do you think is lacking most in PROMs that are developed today? 11. Are there any aspects of the PROM development process which could make PROMs more fit-for-purpose? | - How would you advice researchers to go about making their PROMs more fit-for-purpose? |
|  | Challenges | 12. What are the common challenges that you have faced as a researcher when developing a PROM that is fit-for-purpose? 13. Out of these challenges, is there one that stands out in particular and if so could you outline why you think that is? | - What were the reasons why you and your team faced these challenges? - How do you think that adopting a more intersectional approach may assist or hinder these common challenges? |
|  | Research Team Diversity | 14. Could you tell me a bit about the types of teams that you worked on when developing PROMs in the past, and the impact that the team had on how fit-for-purpose the PROM was that you developed. |  |
|  | Summary | 15. Are there any other factors that influence the development of fit-for-purpose PROMs that you would like to share? |  |
| Overcoming Barriers | How to overcome? | 16. Now that we have discussed some of the challenges involved in developing fit-for-purpose PROMs and intersectional approaches, how do you think that these barriers could be overcome? |  |
|  | Other changes | 17. Are there any other changes that you would like to see in the way in which PROMs are developed? | - What advice would you give to a fellow researcher that would like to develop a PROM that is fit-for-purpose? |
|  | Hypothetical Experience of intersectional PROM development | 18. If you were to develop another COA, would you consider trying to integrate an intersectional approach to PROM development and what would influence this decision? | - [If the researcher would try and integrate an intersectional approach] In what ways might you try and do this and why? |
| Future | Political Climate | 19. What role do you think that the recent political climate will have on the future of PROM development and research on fit-for-purpose PROMs? |  |
| Closing Remarks |  | 20. Is there anything else related to PROM development and/or intersectionality that you would like to share with me – or that you were hoping that I’d ask you about? 21. Is there anything in particular that you would like to ask me? |  |
|  | Thank you very much for offering your time today for this interview. Your participation is much appreciated. If you chose to hear more from me about the findings or about reading the transcripts for redaction then I will be in touch in the next few months. If not, then thanks again and all the best! | | |

**Stop the recording**

**References:**

1. Balsam KF, Molina Y, Beadnell B, Simoni J, Walters K (2011) Measuring Multiple Minority Stress: The LGBT People of Color Microaggressions Scale. Cultur Divers Ethnic Minor Psychol, 17(2): 163-174.
2. Keum BT, Brady JL, Sharma R, Lu Y, Kim YH, Thai CJ (2018) Gendered Racial Microaggressions Scale for Asian American Women: Development and initial validation. Journal of Counseling Psychology 65(5): 571-585.
3. Liu T, Wong YJ, Maffini CS, Goodrich Mitts N, Iwamoto DK (2018) Gendered Racism Scales for Asian American Men: Scale development and psychometric properties. Journal of Counseling Psychology 65(5): 556-570.
4. Schrager SM, Goldbach JT, Mamey MR (2018) Development of the Sexual Minority Adolescent Stress Inventory. Frontiers in Psychology 9:319. doi.org/10.3389/fpsyg.2018.00319.
5. Kerrigan D, Karver TS, Barrington C. Davis W, Donastorg Y, Perez M, Gomez H, Mbwambo J, Likindikoki S, Shembilu C, Mantsios A, Wilson Beckham S, Galai N, Chan KS. (2021) Development of the Experiences of Sex Work Stigma Scale Using Item Response Theory: Implications for Research on the Social Determinants of HIV. AIDS and Behavior 25(2): 175-188.
6. Aldalur A, Pick LH (2022) Development of the Multidimensional Inventory of Deaf Acculturative Stress. J Deaf Stud Deaf Educ 27(4): 408-422.
7. Kalichman SC, Shkembi B, El-Krab R (2023) Geometric approach to measuring intersectional stigma among black sexual minority men: reliability and validity in an HIV prevention context. Sexual Health 20(5): 441-452.
8. Shepherd BF, Maki JL, Zelaya DG, Warner Ş, Wilson A, Brochu PM (2023) Development and Validation of the Gay-Specific Intraminority Stigma Inventory (G-SISI): Initial Evidence Underpinned by Intraminority Stress Theory. Eur. J. Investig. Health Psychol. Educ 13: 170-186.
9. Strang JF, McClellan LS, Raaijmakers D, Caplan R, Klomp SE, Reutter M, Lai MC, Song M, Gratton FV, Dale LK, Schutte A, de Vries ALC, Gardiner F, Edwards-Leeper L, Minnaard AL, Eleveld NL, Corbin E, Purkis Y, Lawson W, Kim DY, van Wieringen IM, Rodríguez-Roldán VM, Harris MC, Wilks MF, Abraham G, Balleur-van Rijn A, Brown LXZ, Forshaw A, Wilks GB, Griffin AD, Graham EK, Krause S, Pervez N, Bok IA, Song A, Fischbach AL, van der Miesen AIR (2023) The Gender-Diversity and Autism Questionnaire: A Community-Developed Clinical, Research, and Self-Advocacy Tool for Autistic Transgender and Gender-Diverse Young Adults. Autism Adulthood 5(2): 175-190.
10. Bradley DL, Kramer CT, Sufrin CB, Scott KA, Hayes CM. (2024) “Because I Was a Criminal and Drug Addict": Experiences of Anti-Black Gendered Racism and Reproductive Injustice Among Black Pregnant and Postpartum Women with a Substance Use Disorder and Incarceration and Family Policing Histories. J Racial Ethn Health Disparities 12: 2204–2220
11. Caldas B, Portela M, Stelson E, Singer S, Amaral T, Amaral C, Escosteguy C, Martins M, de Andrade CLT, Soares L, Cornish F, Rosenthal M, Aveling EL (2024) Promoting equity, diversity, and inclusion in surveys: insights from a patient-engaged study to assess long COVID health-care needs in Brazil. J Clin Epidemiol 173:111423. doi: 10.1016/j.jclinepi.2024.111423.
12. Kambanis PE, Bottera AR, Mancuso CJ, Spoor SP, Anderson LM, Burke NL, Eddy KT, Forbush KT, Keith JF, Lavender JM, Mensinger JL, Mujica C, Nagata JM, Perez M, De Young KP (2022) Eating Disorder Examination-Questionnaire and Clinical Impairment Assessment norms for intersectional identities using an MTurk sample. Int J Eat Disord 55(12):1690-1707.
13. Kalantzis MA, Braden AL, Haidar A. (2024) Disordered eating and emotional eating in Arab, middle eastern, and north African American women. Eat Behav 53: 101868. doi.org/10.1016/j.eatbeh.2024.101868.
14. Cintron DW, Matthay EC, McCoach DB (2023) Testing for intersectional measurement invariance with the alignment method: Evaluation of the 8-item patient health questionnaire. Health Services Research 58(S2): 248-261.
15. Sartor CE, Kennelly N, Chung T, Latendresse SJ (2025) Establishing measurement equivalence of a youth-reported parental monitoring measure across sex, race/ethnicity, and intersectional identity. Journal of Family Psychology 39(2): 160–170.
16. Bowleg L, English D, Del Rio-Gonzalez AM, Burkholder GJ, Teti M, Tschann JM (2016) Measuring the Pros and Cons of What It Means to Be a Black Man: Development and Validation of the Black Men's Experiences Scale (BMES) HHS Public Access. Psychol Men Masc 17(2): 177-188.
17. Scheim AI, Bauer GR (2019) The Intersectional Discrimination Index: Development and validation of measures of self-reported enacted and anticipated discrimination for intercategorical analysis. Soc Sci Med 226: 225-235.
18. Wadsworth LP, Potluri S, Schreck M, Hernandez-Vallant A (2020) Measurement and impacts of intersectionality on obsessive-compulsive disorder symptoms across intensive treatment. American Journal of Orthopsychiatry 90(4): 445-457.
19. Lee EY, Airton L, Jung E, Lim H, Latimer-Cheung A, Szto C, Adams ML, Faulkner G, Gerguson L, Peers D, Phillips S, Yi JK (2024) Development and validation of the SAFE (Socially Ascribed intersectional identities For Equity) questionnaire. Acta Psychologica 245: 104235. doi.org/10.1016/j.actpsy.2024.104235.
20. Lima JD, Plácido J, Andrade B, Abend LD, Waclawovsky AJ, Pires DA, Silva DRPD, Jesus-Moraleida FR, Moura HF, Coelho NLG, Monteiro-Junior RS, Matias TS, Schuch FB, Deslandes AC (2025) Intersectionality and mental health in university students: a jeopardy index approach. Rev Saude Publica 59:e3. doi: 10.11606/s1518-8787.2025059006197.
21. Tong A, Sainsbury P, Craig J (2007) Consolidated criteria for reporting qualitative research (COREQ): a 32-item checklist for interviews and focus groups. International Journal for Quality in Health Care 19(6): 349–357.
22. Glaser B, Strauss, A (1967) The Discovery of Grounded Theory: Strategies for Qualitative Research. Sociology Press, Mill Valley, CA.
23. McCann T, Clark E (2003) Grounded theory in nursing research: Part 1 – Methodology. Nurse Researcher 11(2): 7-18.
24. Charmaz K (2006) Constructing Grounded Theory: A Practical Guide through Qualitative Analysis. Sage, London.
25. Payne S (2007) Grounded theory. In: Lyons E, Coyle A (eds) Analysing qualitative data in psychology. Sage, London.
26. Charmaz K (2012) The Power and Potential of Grounded Theory. Medical Sociology Online 6: 2-15.
27. Handcock MS, Gile KJ (2011) Comment: On the Concept of Snowball Sampling. Sociological Methodology 41(1): 367-371.
28. McGhee G, Marland GR, Atkinson J (2007) Grounded theory research: Literature reviewing and reflexivity. Journal of Advanced Nursing 60(3): 334-342.
29. Braun V, Clarke V (2019) To saturate or not to saturate? Questioning data saturation as a useful concept for thematic analysis and sample-size rationales. Qualitative Research in Sport, Exercise and Health 13(2): 201–216.
30. Ahmed SK (2025) Sample size for saturation in qualitative research: Debates, definitions, and strategies. Journal of Medicine, Surgery, and Public Health 5: 100171. doi.org/10.1016/j.glmedi.2024.100171.
31. Collins PY (2015) Intersectionality's Definitional Dilemmas. Annual Review of Sociology 41: 1-20.
32. Lumivero (2023) *NVivo* (Version 14). [www.lumivero.com](http://www.lumivero.com). Accessed 19 February 2025.

1. Intersectionality offers the: *“critical insight that race, class, gender, sexuality, ethnicity, nation, ability, and age operate not as unitary, mutually exclusive entities, but as reciprocally constructing phenomena that in turn shape complex social inequalities”* [31]. In this sense, intersectionality serves as a framework for understanding how individuals experience inequity multidimensionally.
2. Intersectionality offers the: *“critical insight that race, class, gender, sexuality, ethnicity, nation, ability, and age operate not as unitary, mutually exclusive entities, but as reciprocally constructing phenomena that in turn shape complex social inequalities”* [31]. In this sense, intersectionality serves as a framework for understanding how individuals experience inequity multidimensionally.
